# The State of Mind of Health Care Professionals in Light of the COVID-19 Pandemic: Text Analysis Study of Twitter Discourses

**DOI:** 10.2196/30217

**Published:** 2021-10-22

**Authors:** Aviad Elyashar, Ilia Plochotnikov, Idan-Chaim Cohen, Rami Puzis, Odeya Cohen

**Affiliations:** 1 Software and Information Systems Engineering Ben-Gurion University of the Negev Beer Sheva Israel; 2 Cyber@BGU Ben-Gurion University of the Negev Beer Sheva Israel; 3 School of Public Health Ben-Gurion University of the Negev Beer Sheva Israel; 4 Department of Nursing Ben-Gurion University of the Negev Beer Sheva Israel

**Keywords:** health care professionals, Twitter, COVID-19, topic analysis, emotion analysis, sentiment analysis, social media, machine learning, active learning

## Abstract

**Background:**

The COVID-19 pandemic has affected populations worldwide, with extreme health, economic, social, and political implications. Health care professionals (HCPs) are at the core of pandemic response and are among the most crucial factors in maintaining coping capacities. Yet, they are also vulnerable to mental health effects caused by managing a long-lasting emergency with a lack of resources and under complicated personal concerns. However, there are a lack of longitudinal studies that investigate the HCP population.

**Objective:**

The aim of this study was to analyze the state of mind of HCPs as expressed in online discussions published on Twitter in light of the COVID-19 pandemic, from the onset of the pandemic until the end of 2020.

**Methods:**

The population for this study was selected from followers of a few hundred Twitter accounts of health care organizations and common HCP points of interest. We used active learning, a process that iteratively uses machine learning and manual data labeling, to select the large-scale population of Twitter accounts maintained by English-speaking HCPs, focusing on individuals rather than official organizations. We analyzed the topics and emotions in their discourses during 2020. The topic distributions were obtained using the latent Dirichlet allocation algorithm. We defined a measure of topic cohesion and described the most cohesive topics. The emotions expressed in tweets during 2020 were compared to those in 2019. Finally, the emotion intensities were cross-correlated with the pandemic waves to explore possible associations between the pandemic development and emotional response.

**Results:**

We analyzed the timelines of 53,063 Twitter profiles, 90% of which were maintained by individual HCPs. Professional topics accounted for 44.5% of tweets by HCPs from January 1, 2019, to December 6, 2020. Events such as the pandemic waves, US elections, or the George Floyd case affected the HCPs’ discourse. The levels of joy and sadness exceeded their minimal and maximal values from 2019, respectively, 80% of the time (*P*=.001). Most interestingly, fear preceded the pandemic waves, in terms of the differences in confirmed cases, by 2 weeks with a Spearman correlation coefficient of *ρ*(47 pairs)=0.340 (*P*=.03).

**Conclusions:**

Analyses of longitudinal data over the year 2020 revealed that a large fraction of HCP discourse is directly related to professional content, including the increase in the volume of discussions following the pandemic waves. The changes in emotional patterns (ie, decrease in joy and increase in sadness, fear, and disgust) during the year 2020 may indicate the utmost importance in providing emotional support for HCPs to prevent fatigue, burnout, and mental health disorders during the postpandemic period. The increase in fear 2 weeks in advance of pandemic waves indicates that HCPs are in a position, and with adequate qualifications, to anticipate pandemic development, and could serve as a bottom-up pathway for expressing morbidity and clinical situations to health agencies.

## Introduction

The COVID-19 pandemic has affected nations and societies worldwide. The pandemic became a significant health crisis, with extreme health, economic, social, and political implications. COVID-19 created a unique situation, one which requires that people from different countries, cultures, and life circumstances stand against the same emergency situation [1].

Online social networks may provide insights on the state of mind and the experience of people during COVID-19, from emotional effect [2] to adherence to restrictions [3].

Studies found that negative sentiments were dominant in tweets posted by people during the pandemic [2,4], and Twitter could advance social stigmas in those situations [5]. According to Park et al [6], the spread of information related to COVID-19 on Twitter was faster than in other content networks. They showed that the spillover effect of information that included medical knowledge about COVID-19 was more significant than news with nonmedical content [6]. Many publications relating to Twitter analyses during the pandemic highlighted health care agencies’ and professionals’ critical roles in providing reliable information disseminated via online social networks during intense situations [4,6]. Contrary to studies that analyzed the general population, others focused on specific populations, such as policy makers [7], students [8], and health care professionals (HCPs) [9].

HCPs are an essential resource for public health. The World Health Organization recognized the health workforce as one of the six foundations for improving health outcomes [10] and perceived the development of HCPs as an opportunity for the sustainable development of society as a whole [11]. During an emergency, HCPs are one of the most crucial factors in developing surge capacity to satisfy health care demands [12].

Yet, they are also vulnerable to personal concerns, fear, and anxiety [13] caused by managing a long-lasting emergency situation with a lack of resources and under accelerated conditions. The situation of HCPs struggling to balance day-to-day self-management while having to intensify their work is exacerbated by their circumstances, such as having elderly family members, children at home, or family members with special needs [14].

Most existing studies of the HCP experience during COVID-19 are (1) cross-sectional: demonstrating associations with no causality between study variables, (2) hypothesis driven: unlikely to produce new findings that are not grounded in existing theory, and (3) small scale: study populations range from a few hundred to a few thousand. There are a lack of data-driven longitudinal studies based on large-scale analysis of the HCP experience during COVID-19.

This study aims to analyze the state of mind of HCPs as expressed in online discussions published on Twitter in light of COVID-19 from the onset of the pandemic until the end of 2020. The analyzed data include 16.6 million English tweets from 53,063 HCP accounts identified using a tailor-made machine learning classifier. We present the major topics concerning the study population and the dynamics of emotions during the pandemic.

## Methods

### Identifying the Study Population

**Figure 1:**
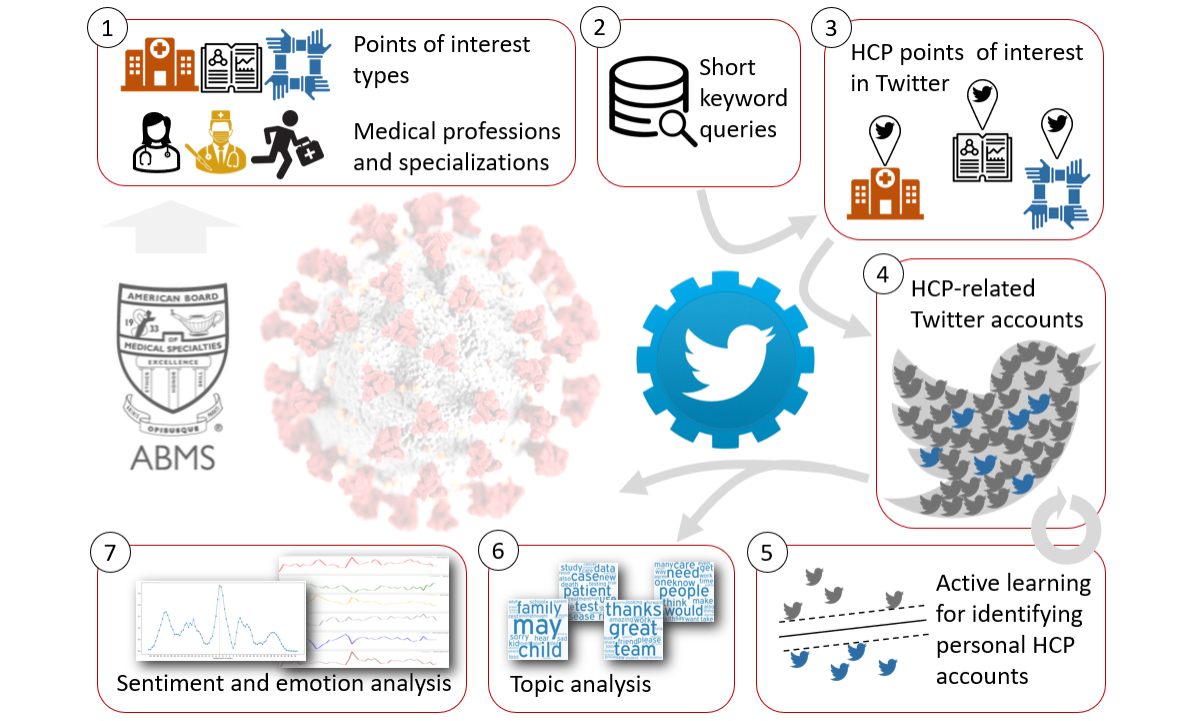
Analysis of the health care professional (HCP) discourse in light of COVID-19. Steps 1 to 5 identify the study population. Steps 6 and 7 analyze the HCP discourse. In step 1, we identify search terms. In step 2, we construct short keyword queries. In step 3, we pinpoint and manually distill HCP points of interest. In step 4, we collect a pool of health care–related Twitter accounts. In step 5, we conduct active learning for filtering out organizational and non-HCP accounts. In step 6, we collect the tweets and analyze the major topics. In step 7, we analyze sentiment and emotions.

This study’s first and significant challenge in this study was to collect tweets published by the heterogeneous HCP population, while excluding formal communication by health care facilities and organizations. We tackled this challenge through a multistep process depicted in Figure 1, steps 1 to 5.

### Twitter Search Queries

First, two health care experts defined a list of general medical professions and specializations recognized by the American Board of Medical Specialties (Figure 1, step 1, and Table S1, list 1, in Multimedia Appendix 1). Second, a list of points of interest (POIs) types, such as unions, conferences, and journals, were defined (Figure 1, step 1, and Table S1, list 2, in Multimedia Appendix 1). In addition, we added countries and regions related to English speakers. We created short keyword queries by taking one keyword from each list, for example, “immunology journal” or “family medicine England” (Figure 1, step 2).

### HCP Points of Interest

#### Overview

We used the Twitter search engine to collect accounts matching the short keyword queries (Figure 1, step 3). The results were manually inspected to identify accounts managed by facilities, organizations, and venues related to health care. The resulting 563 accounts were divided into four groups of medical professions and a fifth group of general health care interest (Table S2 in Multimedia Appendix 1). We removed from this list 41 POIs that are likely to be followed by non-HCPs.

#### HCP Twitter Accounts

Using the Twitter application programming interface, we collected 434,825 followers of the remaining 522 HCP POIs prioritizing those that followed multiple POIs. Twitter accounts collected in this manner included private accounts and accounts managed by organizations, for example, official accounts of medical centers in the United States. There are also many non-HCP accounts, for example, patients, reporters, or academic scholars.

The definition of HCP varies considerably. Broad definitions of HCP consider any professional that contributes to people’s well-being as an HCP. In this study, we define an HCP as an individual working in the health care system or being a student of any medical profession. Students were also included since they typically go through hands-on training that incorporates interaction with patients. Effectively, the medical professionals that were considered HCPs were mainly medical doctors, nurses, medical management and administration staff, pharmacists, medical students, psychologists, and others. We excluded therapists that work in fields that are mostly considered complementary or alternative medicine (eg, naturopaths and shamans) and art therapy (eg, drama and music therapists).

#### Training Machine Learning Classifiers Using Active Learning

To filter out organizational and non-HCP Twitter accounts, we trained two respective machine learning classifiers through an iterative process known as active learning [15]. As done by Lo et al [16], in this research, we used the support vector machine (SVM) classification model. Term frequency–inverse document frequency features were extracted from the accounts’ descriptions and full names. The latter occasionally included a profession or a relevant title, such as “MD” (medical doctor) or “RN” (registered nurse).

Supervised machine learning requires a labeled training set to build accurate statistical models. Active learning allows reduction of the manual annotation effort by focusing on accounts that would contribute the most to improving the classification model. Therefore, we used the uncertainty-sampling active learning strategy [17], where we manually annotated accounts that the classifier is least certain about.

The descriptions and timelines of 90 accounts were inspected by two human annotators in every active learning iteration. In case of a conflict, a third annotator determined the label after an open discussion. In case of uncertainty, the LinkedIn profile associated with the Twitter account was inspected as well. If the panel of three annotators could not agree about the label of an account, it was excluded from the training set and replaced with a random unlabeled account on which the panel had agreed. Every iteration ended with training two SVM classifiers that were used for sampling accounts for the next iteration. Initially, the accuracy of the classifiers increased by 1% to 3% with each iteration. Between the 15th and 20th iteration, the marginal increase in accuracy was as low as 0.5%. Thus, we decided to halt the manual labeling process after 20 iterations (Figure S1 in Multimedia Appendix 1).

Overall, the final training data set contained 1392 profiles on which we performed 10-fold cross-validation for evaluation. The results of this training process are presented in the Results section. Using the trained classifiers, we selected accounts classified as individual HCPs with a confidence of 70% or higher and validated the quality of this selection by manual inspection of 100 random accounts.

### Social Discourse: Topic Detection

We collected all public tweets published by the individual HCP accounts from January 1, 2019, to December 6, 2020; see Table 1 for details about the data set of collected tweets.

We used Python (version 3.6.8; Python Software Foundation) and several libraries for our analysis. We applied standard text preprocessing techniques, such as removing line breaks, emojis, nonalphabetic words, stop words, short words with less than three characters, user mentions (@), and hyperlinks; we applied lower-casing and lemmatization using Natural Language Toolkit (NLTK) (version 3.5; NLTK Team) [18] and WordNet (version 3.0; Princeton University) [19]. Hashtag (#) terms were not removed because they carry a significant informative load on Twitter. The hashtag symbol itself was removed.

Most of the social discourse during 2020 revolved around COVID-19. This theme overshadowed other topics discussed both with and without a relationship to the pandemic. Therefore, to have a precise dissection of topics discussed during 2020, we removed the terms signifying the disease or the virus. The full list of COVID-19 terms can be found in Table S3 in Multimedia Appendix 1.

**Table 1:** The study data set.

Statistic	Value
Accounts, n	53,063
Tweets, n	16,616,970
Tweets, mean (SD)	313 (1386.6)
Friends, mean (SD)	511 (1767.1)
Followers, mean (SD)	475 (4466.8)
Total tweets published in 2019, n	7,168,088
Total tweets published in 2020 (up to December 6, 2020), n	9,448,882

Topic models were obtained using the latent Dirichlet allocation (LDA) algorithm [20] implemented in the gensim library (version 3.8.3; Python Software Foundation) [21]. The number of iterations was set to 150 with a chunk size of 130,000 tweets. All other parameters were set to their default values. We searched for the optimal number of topics between 5 and 55. To quantify the quality of the topic distributions, we used one of several coherence scores that were proposed by Roder et al [22]. In their work, human judgments of the interpretability of topics extracted from several benchmark data sets were recorded. The authors then examined the correlation of each measure against the human interpretability scores. We chose the coherence score, which is the most correlative to human topic rankings. In addition to coherence scores, we also examined the Jaccard similarity index between the sets of top 100 words of topics originating in different distributions using the multiple correspondence analysis algorithm (configured with px=py=0.95) from the Jaccard package (version 0.1.0) in the R language (version 4.1.0; The R Foundation). To determine the topics that persist in different distributions, we studied the connections between distributions to eventually decide on the best one regarding the number of similar topics that it shares with other distributions.

Provided the topic distributions, volumes of the topics, and their persistence over different distributions, we chose to analyze the topic distribution containing 20 topics. A manual subjective inspection was conducted to make sense of the automatically generated topics. The manual inspection included an assessment of the topics’ subjective cohesion level and topic naming. Each topic received a subjective cohesion score based on our ability to name the topic. The naming relies on the top 50 words within the topic and the contents of a few hundred tweets with the highest probability of belonging to the topic. There are three cohesion levels: high, medium, and low. A high cohesion score was given to topics where most of the 50 top words could easily be associated with a single well-defined theme, and most of the inspected tweets matched this theme. A medium cohesion score was given to a topic when some of the 50 top words could easily be associated with a specific theme, yet a relatively high ratio of words and tweets could not be associated with it. In cases where no single name could be identified that described a significant number of the topic’s 50 top words and top tweets, the topic received a low subjective cohesion score and no name.

We chose the main topics for further analysis based on their volumes, coherence, and cohesion scores. Weekly volumes of the chosen topics were tracked throughout the year 2020. We identified the major changes in topic volumes and associated them with the most significant events presumably corresponding to the changes in topic volumes. The topic analysis results are discussed in the Topic Detection subsection within the Results section.

### Analysis of Sentiment and Emotions

To estimate the sentiments expressed by HCPs, we used the Valence Aware Dictionary and Sentiment Reasoner (VADER), which is a lexicon and rule-based sentiment analysis tool [23]. Given a tweet, VADER provides a sentiment score, which ranges from –1 (the highest negative score) to 1 (the highest positive score). To track the changes in sentiment, we calculated the average sentiment of each topic every week, as well as the Bayesian credible interval of 95% to ensure that the average sentiment was a good representative of the sample obtained.

Sentiment is a coarse-grained measure that does not allow for understanding the different emotional tones expressed in text. Therefore, we used a pretrained recurrent neural network model developed by Colnerič and Demšar [24] for quantifying the probabilities of Ekman’s six basic emotions [25] expressed in the text. We inferred the distribution of emotions in a topic by aggregating the emotion probabilities across all tweets associated with the topic. We included retweets and tweets containing quotes when aggregating emotions and sentiment, since by retweeting or quoting an emotionally loaded tweet, a person shows their support for it. Differences of emotions between the years 2019 and 2020 were calculated based on the emotion distribution: the Welch *t* test for normally distributed emotions (ie, anger, sadness, and joy) and the Mann-Whitney *U* test for nonnormally distributed emotions (ie, fear, surprise, and disgust).

Next, we analyzed the time course of each emotion and quantified their correlation during the study periods (47 weeks) with the following: number of new COVID-19 cases (*Confirmed*), number of deaths caused by COVID-19 (*Deaths*), their weekly change (*∆Confirmed* and *∆Deaths*, respectively), and the estimated reproduction rate of SARS-CoV-2 (*R_t_*). A Shapiro-Wilk test was conducted to examine the distribution of the variables. Cross-correlation analysis was performed to account for possible lags in a range of 1 to 8 weeks between pandemic development and emotional response. This study was approved by the Institutional Review Board of Ben-Gurion University of the Negev (1879-1).

## Results

### HCP Accounts on Twitter

During the active learning process, we manually labeled 1800 Twitter accounts. Out of these accounts, 1192 (66.2%) were labeled as profiles of individuals and 299 (16.6%) were labeled as organizational. The best classifier differentiated between individuals and organizations with an accuracy score of 0.88, an F1 score of 0.88, a precision score of 0.884, and a recall score of 0.88. The best classifier, trained on accounts labeled as individuals to take HCP and non-HCP accounts apart, obtained an accuracy score of 0.786, an F1 score of 0.785, a precision score of 0.795, and a recall score of 0.787. Performance scores are reported for balanced test sets with subsampling of the majority class.

Out of the 434,825 HCP POI followers, 53,063 profiles were classified as individual HCPs with a confidence of 70% or higher. Random manual validation of 100 accounts confirmed that 90 of these accounts belonged to health care individuals.

### Topic Detection

**Figure 2:**
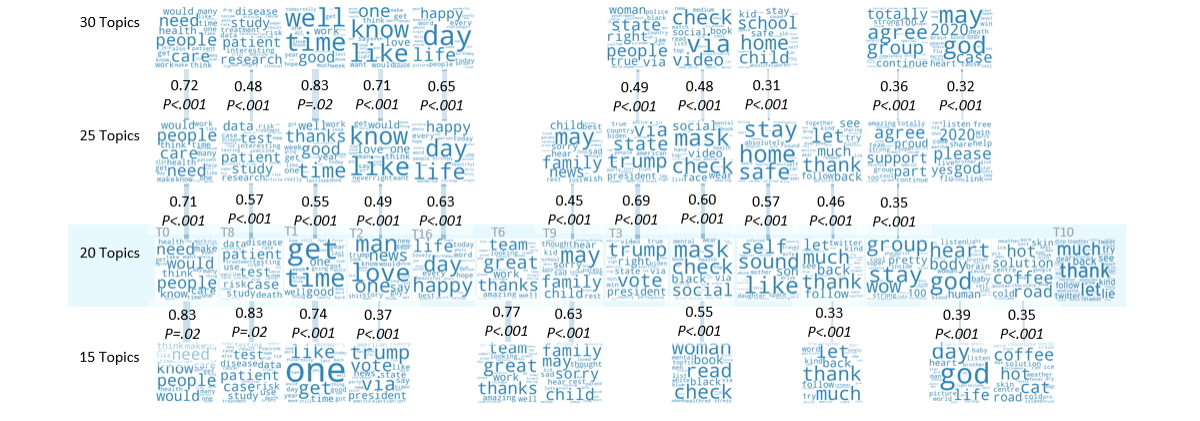
Alignment of topic distributions. Topics, represented with word clouds, were obtained from four different distributions of 15, 20, 25, and 30 topics. Every pair of aligned topics is connected using a weighted link. The weight and the width of each link represent the Jaccard coefficient of the sets of words associated with the two aligned topics; coefficients are reported in the top rows of each set of values. Links having a Jaccard coefficient lower than 0.3 are not shown. Topics that have no link are also not shown (except topic 10).

The LDA algorithm’s topic detection resulted in the highest average coherence of 0.433 for the distribution of 30 topics. The distributions of 25 and 20 topics exhibited average coherence scores of 0.427 and 0.402, respectively. The coherence values for each topic distribution were normally distributed. We linked topics in different distributions based on the Jaccard coefficient of the sets of tweets associated with the topics. As can be seen in Figure 2, there are no topics linked to more than one topic in a different distribution with a Jaccard coefficient higher than 0.3. The alignment of topic distributions also shows that four topics persisted throughout distributions of 15, 20, 25, and 30 topics. These topics were “public health and social values” (topic 0), “day-to-day life” (topic 1), “food” (topic 2), and “medical studies and COVID-19 information” (topic 8). Topics 0, 1, and 2 contained the largest number of tweets during 2020: 25.9%, 27.7%, and 13.5%, respectively.

**Figure 3:**
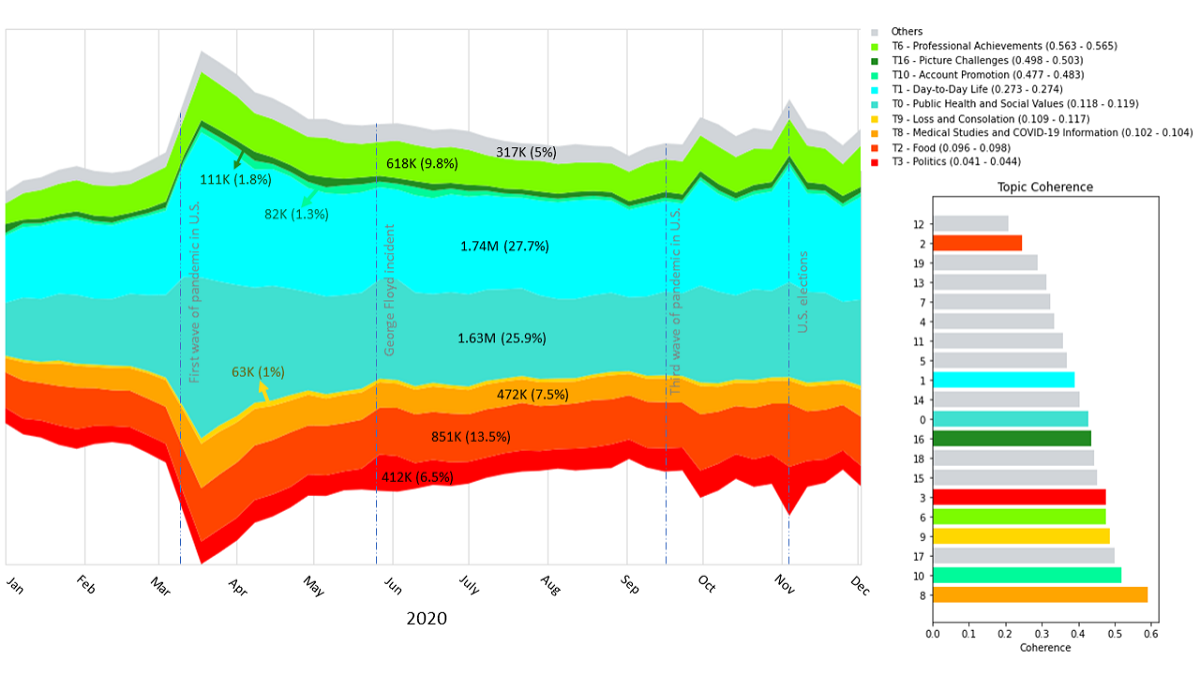
Topic (T) volumes over time in 2020 sorted by sentiment and their coherence. The 95% CIs of the sentiments are provided in parentheses alongside the topic names in the legend.

Topic 8 (“medical studies and COVID-19 information”) also contained a significant fraction of tweets (7.6%) and had the highest coherence score in the distribution of 20 topics (Figure 3). The distribution of 20 topics contained the highest number of persistent topics (n=14).

Topic volumes are provided in thousands or millions of tweets over the entire period. The percentage of the total volume is provided in parentheses. Topics are sorted and color coded according to the average sentiment in the main chart and the legend. The 95% CIs of the sentiments are provided in parentheses alongside the topic names.

Further manual inspection of the distribution of 20 topics revealed that the most cohesive topics were also the most persistent, except topic 10 (“account promotion”), which received a high cohesion score but appeared only in this distribution. This topic accounted for 1.8% of the tweets. Overall, topics 0, 1, 3, 6, 8, 9, 10, and 16 received high cohesion scores and are listed in Table 2.

**Table 2:** The most significant topics discussed by health care professionals during the COVID-19 pandemic.

Title	Example tweet	Explanation	Top 25 words^a^
Public health and social values (topic 0)	“Medicare being defunded for primary care services. There’s a huge opportunity to reform healthcare to invest where the best evidence sits. Primary care & preventive activities. (Public health too cos investment in public housing education & early life is essential part of this)”	Tweets on this topic discuss issues of public health policy and its applications as well as social values that are mostly related to health aspects.	People, need, would, know, think, care, make, health, many, one, get, time, take, want, work, even, way, agree, thing, also, help, say, right, public, doctor
Day-to-day life (topic 1)	“10 years ago I was sitting on the sofa waiting to watch Jools Holland’s Hootenanny. Tonight, I am sitting on the sofa waiting to watch Jools Holland’s Hootenanny. In ten years time, I hope to be sitting on the sofa waiting to watch Jools Holland’s Hootenanny. #Hootenanny”	This topic contains tweets that mainly describe typical everyday situations.	Get, time, good, one, well, year, got, going, know, week, last, thing, back, really, still, done, work, would, home, see, think, yes, look, first feel
Food (topic 2)	“This is so delicious and easy. I have been eating and making it for 35 years..... 4oz (125g) blanched almonds, toasted 4oz cherries 2oz mixed candied peel 2oz raisins 2oz sultanas 2oz currants 1 des sp mixed spice 1 tsp cinnamon 1 tsp nutmeg”	This topic consists of tweets about restaurants, impressions from dishes, recipes, and other food-related matters.	Love, man, one, news, say, trump, story, guy, know, new, would, shit, game, look, show, think, eat, ever, via, song, want, make, food, said, house
Politics (topic 3)	“I want my President to respect Americans. Republican Americans, Democrat Americans, members of our American military, our democracy, women, law and order, the Constitution, our country...”	The tweets on this topic are generally related to politics and government, particularly to the United States’ contemporary affairs.	Trump, vote, president, right, state, via, video, true, win, biden, party, sign, country, police, election, american, america, sir, law, watch, india, funny, stop, joe, republican
Professional achievements (topic 6)	“Proud of all the amazing accomplishments of our fellowship graduates Deputy Medical Director of [hospital], will be presenting at EMSWorld (EMS - EMT - Paramedic Emergency Medical Services) Expo.”	This topic contains tweets that commend teams and individuals for professional accomplishments.	Great, thanks, team, please, work, amazing, friend, looking, congratulation, forward, proud, student, support, share, today, colleague, sharing, fantastic, join, new, welcome, awesome, look, help, brilliant
Medical studies and COVID-19 information (topic 8)	“Nice article on Remdesivir & renal dysfxn. Interesting a 117% (over 2x) increase in end of therapy AKI occurred in pts w/ renal dysfxn was not statistically significant (5% vs 2.3%). Stats are so... interesting... sometimes similar numbers (sample size?) result in FDA approval.”	The tweets on this topic are primarily about science communication of medical studies and epidemiological information related to COVID-19.	Patient, case, test, study, data, risk, disease, use, death, testing, treatment, vaccine, new, rate, surgery, cancer, result, care, also, interesting, number, via, symptom, trial, infection
Loss and consolation (topic 9)	“Sad to hear May Allah rest him in peace and give you and family Sabar! Ameen”	Tweets on this topic convey sorrow and are meant to console individuals and families for the loss of loved ones.	May, family, child, sorry, hear, thought, kid, sad, rest, parent, school, young, loss, loved, sending, thinking, peace, memory, adult, indeed, dad, prayer, anxiety, mom, soul
Account promotion (topic 10)	“Active? Drop your username and retweet lets follow each other Grow together ..FOLLOW BACK.. follow back immediately..No Lie.. Try me”	This topic essentially contains tweets meant to promote accounts and tweets with information about accounts’ activities on Twitter.	Thank, much, let, back, follow, twitter, see, kind, together, tweet, lie, try, dear, drop, retweet, appreciate, active, mention, reach, immediately, grow, following, fan, week, reply
Picture challenges (topic 16)	“Day 5/7, I’ve been challenged by [user mention] to produce a picture a day for 7 days to illustrate my current life (no captions, no people). Nominate a person a day, asking to copy these words, add photo and repeat the challenge. Today I nominate [URL]”	This topic’s tweets are mainly a response to an online social media challenge that prompted Twitter users to post photographs that represent their lives.	Day, happy, life, every, word, today, best, hope, lovely, beautiful, birthday, wish, photo, person, picture, challenge, year, new, save, people, one, enjoy, posted, single, wonderful

^a^Words are listed in order of their prevalence.

### Analysis of the Discussion Topics

#### Content of Discourse

We identified 9 out of 20 topics (45%) that constituted 95% of the total discourse. Table 2 presents the final 9 topics that describe the HCPs’ discussions during the year 2020. For each topic, the top 25 words are presented alongside a short explanation and a representative tweet. The titles of the topics were chosen to match the majority of manually inspected tweets. The tweets in the selected topics discussed both professional (45.5%) and personal (54.5%) issues. All professional topics presented as high coherence levels as the topics of “professional achievements” and “medical studies and COVID-19 information.”

Out of the 9 topics, 8 (89%) received a high subjective cohesion score, while 1 topic (11%; topic 2: “food”) received low coherence and medium cohesion scores. The cohesion of this topic was set to medium because the top 50 words were loosely associated with a common theme. Still, when examining the topic’s tweets, we found that the vast majority of them were related to food. Note that types of food were not discernible in the topic’s top 50 words due to the high diversity of the food types used in the tweets (eg, salad, chicken, and BBQ). The volume of the food topic remained consistently high (13.5%) throughout the year 2020.

The distribution of tweets across HCPs (Figure S2 in Multimedia Appendix 1) was a long-tailed (power-law) distribution. However, the user that tweeted the most accounted for only 0.57% of the data, and the top 60 users accounted for 10%. To check the influence of the top users on the social discourse, we compared their topic distribution to that of the rest of the users (Table S4 in Multimedia Appendix 1). The topic distributions were highly correlated (Spearman *ρ*[9 pairs]=0.9, *P*<.001), showing no significant influence of the top users on the social discourse.

#### Emotion Analysis of the Detected Topics

**Figure 4:**
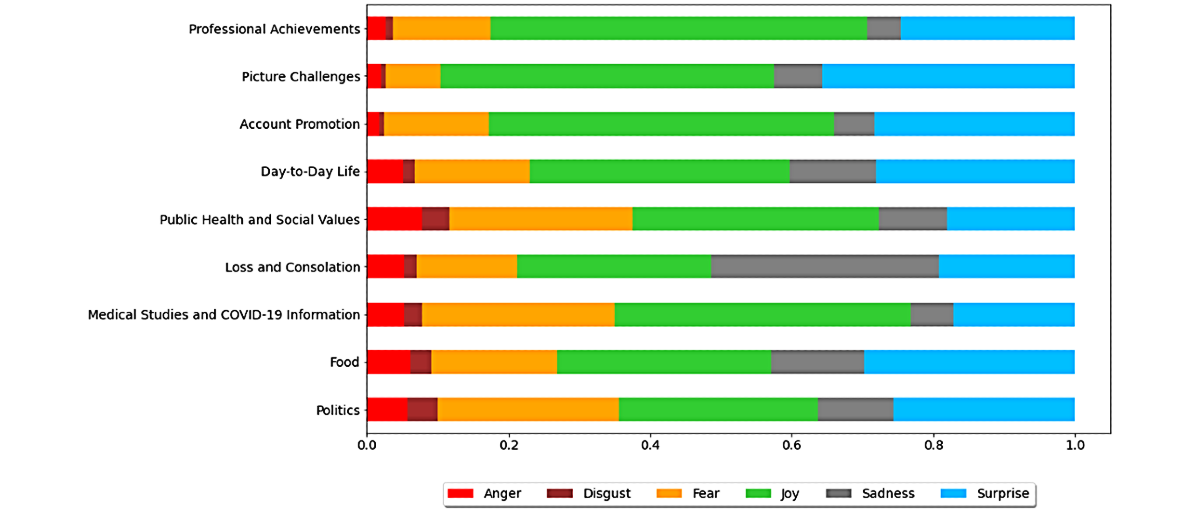
Distribution of emotions per topic.

We computed the average levels of six emotions in each topic: anger, disgust, fear, joy, sadness, and surprise. Topics with the highest levels of joy (about 50%) were “professional achievements” (53.1%), “account promotion” (48.8%), and “picture challenges” (47.0%). As expected, the “loss and consolation” topic presented the highest average sadness (32%) in comparison to the average of 8.6% in the other topics. Similarly, the topic of “politics” presented the lowest ratio of joy and was also the topic with the lowest sentiment score (Figure 4).

#### Trends of Discourse Throughout the Year 2020

Figure 3 describes the topic volumes over time in 2020 sorted by average sentiment and their coherence. It includes the Bayesian credible interval of 95% for each topic’s average sentiment. In addition, the distribution of the sentiment scores for each topic can be found in Figure S3 in Multimedia Appendix 1. Among the identified topics, the “professional achievements” topic presented the highest positive sentiment score of 0.56, while the “politics” topic obtained the lowest sentiment score of 0.04. Trends of discourse during the year 2020 revealed that HCPs express in their discussions special events that have occurred, most of them in the United States. The increase in tweets responded to the global crisis, ahead of the first wave of the COVID-19 pandemic in the United States. Analyzing modifications from the content point of view, the topic of “public health and social values” (topic 0) exhibited the highest increase. “Medical studies and COVID-19 information” (topic 8), together with “day-to-day life” (topic 1), achieved relatively moderate growth. In a milder tendency, these topics reacted similarly to the third wave of the pandemic. The case of George Floyd sparked political (topic 3) discussions at the expense of day-to-day life (topic 1), with a minor impact on the context of public health. Around the US elections, discussions on political issues rose sharply with the reverberation of the elections in the public health topic.

### Emotion Dynamics of the HCPs

#### Overview

**Figure 5:**
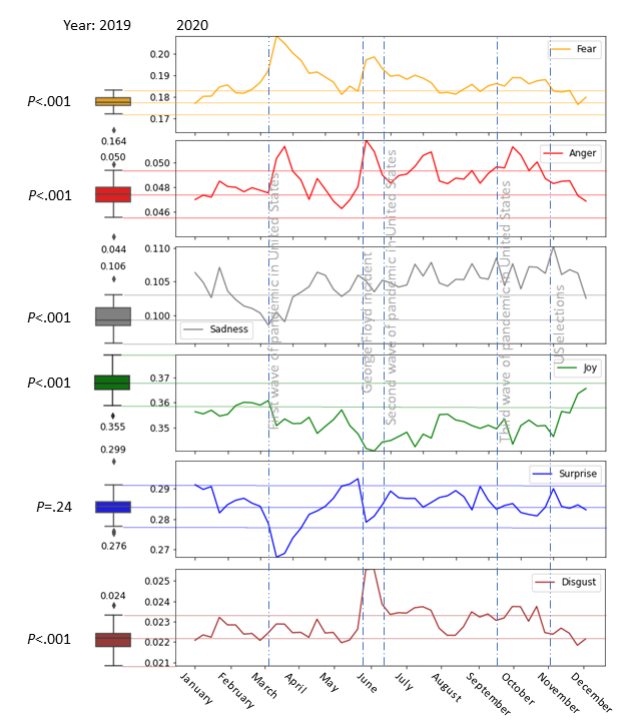
Emotions over time. On the left of the time series, the box plots show the statistics of the year 2019 for each emotion. The boxes represent the IQRs, and the whiskers represent the upper and lower quartiles. The dashed gray vertical lines indicate the important events during 2020. The time series detail the average weekly emotions during 2020. The y-axes represent the intensity of six emotions and are the same for the time series and the box plots. *P* values on the left side refer to the significance of the difference between the emotion levels in 2019 and 2020.

We analyzed the average weekly emotion values in the HCP Twitter discourse during 2020. Figure 5 presents the dynamics of Ekman’s six basic emotions: anger, joy, fear, sadness, surprise, and disgust. We analyzed the sentiment dynamics as well. Since sentiment closely follows the values of joy, we did not present it in Figure 5.

#### Acute Effects

**Figure 6:**
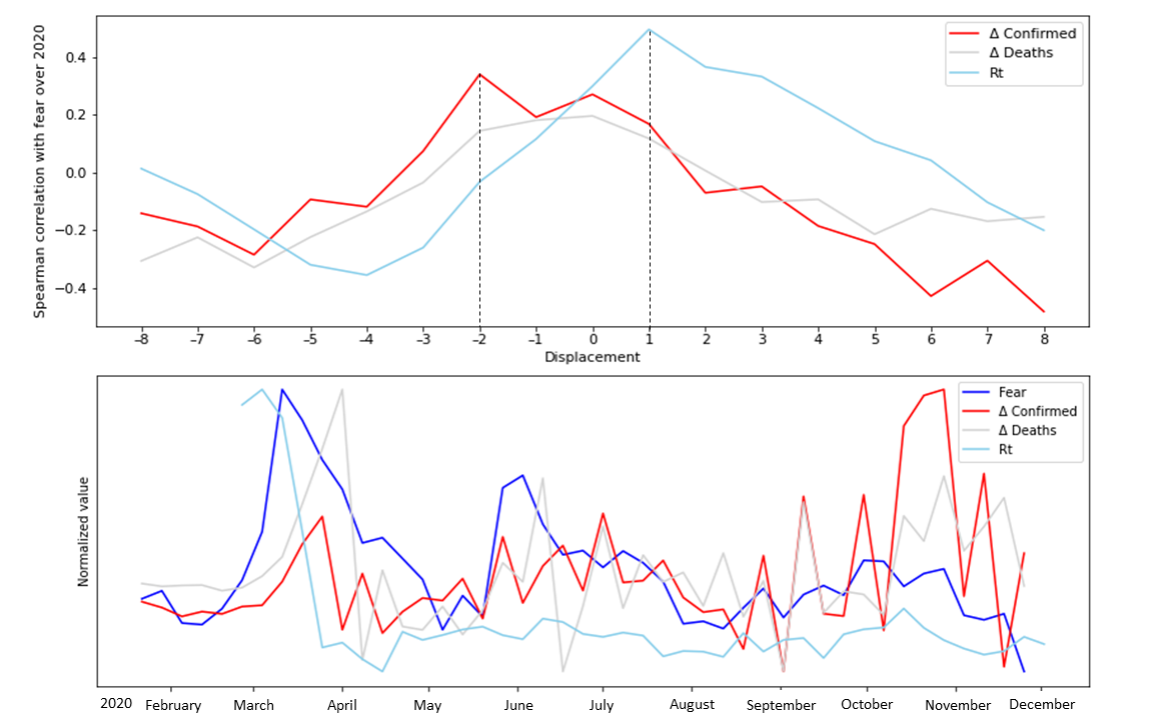
Normalized weekly fear levels (dark blue) during 2020 vs the SARS-CoV-2 reproduction rate (*R_t_*; light blue), the difference in the average number of daily death cases (*∆Deaths*; gray), and the difference in the average number of daily new confirmed cases (*∆Confirmed*; red). The top panel shows the cross-correlation results with displacement ranging from –8 (before fear) to +8 (after fear) in terms of Spearman correlation coefficients.

Tracking the emotions over time showed that anger, fear, and disgust exhibited fluctuations that corresponded to the three COVID-19 pandemic waves worldwide (Figure 5). Fear exhibited the three clear waves, with the first wave being the largest and the second and third magnitudes gradually decreasing. Cross-correlation analysis revealed that fear was found to correlate most highly with the virus reproduction rate *R_t_* with a delay of 1 week (*ρ*[47 pairs]=0.486, *P*=.001). A lesser but significant correlation (*ρ*[47 pairs]=0.340, *P*=.03) was found between fear and *∆Confirmed*, where fear preceded *∆Confirmed* by 2 weeks. The normalized values of fear, *R_t_*, *∆Deaths*, and *∆Confirmed* are shown in Figure 6. The average weekly levels of anger were correlated (*ρ*[47 pairs]=0.386, *P*=.009) with the change in the average number of death cases (*∆Deaths*), with *∆Confirmed* (*ρ*[47 pairs]=0.308, *P*=.04), and with the virus reproduction rate *R_t_* (*ρ*[47 pairs]=0*.*316, *P*=.04) after displacement of 2 weeks (anger after *R_t_*). A positive correlation was found between sadness and *∆Confirmed*, where sadness preceded *∆Confirmed* by 3 weeks (*ρ*[47 pairs]=0.423, *P*=.005). A negative correlation arose between joy and *∆Confirmed,* where joy decreased after 1 week. Two periods of increased disgust roughly corresponded to the second and the third pandemic wave. Interestingly, disgust remained at an average level in the HCP discourse during the first wave, in contrast to fear and anger. Disgust and anger spiked after the George Floyd case and the second wave of the pandemic.

#### Long-term Effects

We can see in Figure 5 that joy was low throughout 2020. Average weekly joy during 2020 was lower than the minimal weekly joy during 2019. We also saw a rise in joy by the end of 2020, bringing it closer to the values observed during 2019. Sadness increased during 2020, with the minimal sadness between April and December 2020 being higher than the maximal sadness before December 2019. The intensities of all emotions, except surprise, were significantly different during 2020 than in 2019 (*P*<.001).

## Discussion

### Overview

HCPs are at the core of pandemic response, and the impact of emergencies, especially a pandemic, on the frontline workforce was found to be significant during former events [26]. This study was designed to explore the state of mind of HCPs in light of the COVID-19 pandemic, as expressed in Twitter discussions during the year 2020. The study was conducted by a multidisciplinary group of researchers from data and health sciences to reflect professional health aspects and data-driven approaches throughout the study. Beyond the results presented in this paper, the study tries to serve as documentation of the broad reality of HCPs during the pandemic. Such documentation was found to be missing during former pandemics, such as the influenza pandemic of 1918-1919 [27].

### Principal Findings

This study explored three principal results:

1. A rigorous multistep process of selecting the study population, which involved active machine learning and manual curation, resulted in a high-quality data set of English tweets from 53,063 HCPs.
2. Analysis of the topics discussed by the HCPs during the year 2020 revealed that Twitter serves as a platform for expressing both day-to-day and professional content during the pandemic. The analyses revealed that discussions could be attributed to events that occurred. As such, the volume of tweets increased appropriately with the progression of the COVID-19 outbreak, and the analyzed data articulated events with influential aspects, such as the pandemic waves and the George Floyd case.
3. Analysis of emotions expressed in Twitter explored the significant emotional impacts of the COVID-19 pandemic on HCPs that were sharpened compared to emotion levels found in 2019. The results portrayed significant long-term and acute responses to the pandemic.

### Comparison With Prior Work

#### Methodological Aspects

Recently, many studies examined different aspects of the COVID-19 pandemic using public information published within social media. Some of the studies attempted to predict the number of reported cases associated with the COVID-19 pandemic based on posts published on Sina Weibo [28,29] or Twitter [30]. Multiple articles analyzed online discourses, sentiments, and general dynamics during the pandemic [4,31-35]. These studies made their conclusions based on posts, which were published in the early stages of the pandemic (ie, over a few months), as opposed to this study, which analyzed online discourses during a single year of the pandemic, including a comparison of the emotional dimension during 2020 with the emotional dimension a year before the pandemic emerged. In addition, most of the studies collected posts using predefined COVID-19–related keywords [31,33] or hashtags [34]. However, it is important to understand the overall state of mind of HCPs during the crisis, including aspects not directly related to the COVID-19 pandemic. Therefore, in this study, we analyzed posts of HCPs across a variety of topics. Although many studies investigated COVID-19 health aspects, only a few of them focused on HCPs.

Similar to this study, a few articles focused on the HCP population [9,32]. Ojo et al [9] addressed a specific research question comparing the COVID-19–related social media reaction of HCPs to gun violence. Wahbeh et al [32] studied a small-scale population of HCPs. The limited scope or population of related work could be explained by the difficulties in identifying HCPs as a study population in social media.

#### Social Media as a Community in Times of Crisis

Studies found that virtual communities provide their members a sense of community, especially in emergencies [36]. According to Neubaum et al [37], in a time of crisis, social media platforms serve as a space for social sharing of emotions and pursuing empathetic concerns. Twitter is currently the most popular form of social media used for health care communication [38]. Moorhead et al [39] recognized social media as a dimension of health care and a powerful tool that offers collaboration between users and a social interaction mechanism for a range of individuals.

Current studies call for HCPs to join the social media space, take part in relevant conversations, and increase their involvement in providing professional information [4,40]. As far as we know, their involvement was not measured. Ojo et al [9] found that tweets from HCPs were more positive and action oriented in the context of the COVID-19 than tweets that dealt with gun violence, but their study addressed only two issues in a short-term period.

Analyzing the topics discussed on Twitter by HCPs during 2020 revealed two interesting findings:

1. About 44.5% of the topics addressed by HCPs during the COVID-19 pandemic were related directly to their professional context (“public health and social values” [26%], “professional achievements” [10%], “medical studies and COVID-19 information” [7.5%], and “loss and consolation” [1%]).
2. Most of the volume of discussions that increased following the pandemic’s waves was related to professional aspects. Although we did not measure the involvement of HCPs in social media during the COVID-19 pandemic, our findings could indicate the role of HCPs in the social media expanse.

HCPs’ discussions on Twitter during the study period showed that HCPs maneuver between their day-to-day reality and their professional aspects in the virtual space. The emotion analysis of the topics (Figure 4) fit the topic themes, validating their appropriateness. For example, “professional achievements” exhibited the highest joy, while “loss and consolation” exhibited the highest sadness. Based on the topic analysis (eg, Figure 2), four topics constituted a solid, persistent part of the HCP discourse unaffected by sampling and the stochastic nature of the topic detection algorithms: “public health and social values” (topic 0), “day-to-day life” (topic 1), “food” (topic 2), and “medical studies and COVID-19 information” (topic 8).

Various studies investigated social media concerning the rise of the COVID-19 pandemic [2]. Several studies analyzed the discussions of public users within social media. Saleh et al [41] attempted to understand public perception of COVID-19 social distancing on Twitter, and Xue et al [42] analyzed users’ discourse and psychological reactions to the pandemic on Twitter. Other studies focused on specific populations, for example, US governors and presidential cabinet members [7], the social media activity and mental health of students in Switzerland [8], detection of users who were found to suffer from depression using transformer-based deep learning models on the Twitter platform [43], among others. Recently, Ojo et al [9] examined the behavior of health care workers on social media concerning two specific public health crises—the COVID-19 pandemic and gun violence—using analysis of two online discussions derived from two selected hashtags.

#### Sentiment and Emotional Effects Among HCPs During the COVID-19 Pandemic

Many studies published during the COVID-19 pandemic assessed the emotional effects of the pandemic on frontline HCPs. Most of these studies were cross-sectional, used questionnaires, and were disseminated through social media platforms. In general, anxiety, stress, and posttraumatic stress disorder were identified among HCPs working in different countries, such as Singapore and India [44], Spain [45], and Italy [46]. Some of the studies revealed that mental health symptoms, such as depression, anxiety, and stress, are associated with the presence of physical symptoms [44].

This study is different, since it did not begin with a known theory framework but was designed as a data-driven exploration of the emotional status of HCPs during the COVID-19 pandemic. Figure 5 shows trends of emotions over time, with comparison to patterns explored in 2019. The apparent difference in all emotions, except surprise, expressed during 2020 as compared to 2019 confirmed the results of previous studies, this time at a large scale in the social media expanse.

We recognized two pathways: (1) acute emotional response to COVID-19 progression and (2) long-term effects (ie, structures of emotions developed over time beyond the direct association with the pandemic waves). Acute responses (ie, anger, fear, and disgust) were associated with the pandemic waves, portraying a singular trend for each emotion. It is interesting to see the differences between the emotions: fear exhibited a decreasing trend over time, although the overall pandemic impact was increasing. This trend and the positive correlation between fear and the development of the reproduction number (*R_t_*) was also found among the general population [47]. Still, the fact that the increase in fear expressed by HCPs preceded the change in confirmed cases may indicate that HCPs express their feelings according to the population behavior they observe. These results emphasize the fact that emotions (eg, fear) among HCPs could be an indicator of the current situation and the near-anticipated future. The results of this study imply that beyond the traditional role of HCPs in providing reliable information to the population, they may also serve as a bottom-up pathway for expressing morbidity and clinical situations to health agencies. Therefore, we suggest that decision makers invest additional resources into listening to the HCP community in the broadest sense, expanding beyond epidemiology professionals. Brief surveys, 1- to 2-minute interviews at workplaces, and online social media analyses may be good sources of such elicitation.

Although this study did not measure the effect of COVID-19 on HCPs’ mental health, other studies explored the correlation between fear and mental health impacts, such as anxiety, stress, and depressive symptoms [48,49]. Based on the study of Braquehais et al [50], the high prevalence of anxiety and depressive symptoms among HCPs were developed due to the exposure to COVID-19 aspects, material and mental resources, and personal factors. Following former studies regarding the effects of the pandemic on the risk behavior of HCPs in the postpandemic period, our results describe the accumulated sadness and the decreasing joy over the year 2020. These findings should be an additional warning sign to health organizations regarding the immense importance of providing available and accessible mental health support to HCPs, assisting them in coping with the pandemic’s consequences.

### Limitations

Our findings should be considered while bearing several limitations. The analyses did not account for the voice of HCPs who do not use the Twitter platform. In addition, we did not compare the discussions of HCPs to the general population’s discussions to explore similarities and differences. This study described the emotional status of HCPs as expressed in their Twitter discussions, unconfirmed by questionnaires or interviews. The correlations presented in this paper do not imply causality. Yet, the correlation between fear and epidemic measurements was confirmed by another empirical study [47] and theory [51].

Recent works showed that geographical differences [52,53], seasonality, and mass media [54] can influence social media discourses. However, in this study, we assumed that HCPs worldwide were exposed to similar conditions during the pandemic and had similar professional backgrounds and training. Hence, we considered the HCPs as a single study population without addressing cultural and geographical differences. Further studies should focus on local circumstances and cultural aspects of each location, seasonality, and the effects of mass media.

### Conclusions

HCPs are at the core of pandemic response, and the impacts of the pandemic were found to have severe mental health and risk behavior implications during former events. A rigorous multistep process of selecting the study population, which involved active machine learning and manual curation, resulted in a high-quality data set of English tweets from 53,063 HCPs.

Analyses of longitudinal data over the year 2020 revealed that about 44.5% of Twitter discussions from HCPs were directly related to professional content. The rise in discussions following the pandemic waves were mostly focused on professional content. Exploring emotional trends expressed in Twitter discussions showed that the emotional realm of HCPs was affected during the COVID-19 pandemic. Thus, it may indicate the utmost importance in providing emotional support for HCPs to prevent fatigue, burnout, and mental health disorders in the postpandemic period.

In addition, the results clearly showed that fear and other emotions in the HCP discourse carried the signal reflecting the current situation and the near-anticipated future. Therefore, decision makers should invest resources into listening to the HCP community in the broadest sense expanding beyond epidemiology professionals. Brief surveys, 1- to 2-minute interviews at workplaces, and online social media analyses may be good sources of such elicitation. Also, the increase in fear 2 weeks before the pandemic waves (*∆Confirmed*) indicated that HCPs were in a position, and with adequate qualifications, to anticipate the pandemic development. Future research directions could include identifying and examining the major factors leading to fatigue and burnout among HCPs using machine learning techniques. Also, recommendations for preventing these adverse effects could be helpful in improving HCPs’ experience in the face of long-term emergencies like a pandemic.

### Script Availability

The scripts used for the analysis, as well as those used to create the figures for the paper, are available in Multimedia Appendix 2 and on GitHub [55].

## Appendix

Multimedia Appendix 1 Supplementary figures and tables.

Multimedia Appendix 2 Scripts that were used for analysis and to create the figures for the paper.

## Abbreviations

HCP: health care professional
LDA: latent Dirichlet allocation
MD: medical doctor
NLTK: Natural Language Toolkit
POI: point of interest
RN: registered nurse
SVM: support vector machine
VADER: Valence Aware Dictionary and Sentiment Reasoner
