# Supplementary material for: The State of Mind of Health Care Professionals in Light of the COVID-19 Pandemic: Text Analysis Study of Twitter Discourses
Source: J Med Internet Res. 2021 Oct 22;23(10):e30217. doi: 10.2196/30217 (PMC8544741; doi:10.2196/30217)
Supplement: Multimedia Appendix 1 [file jmir_v23i10e30217_app1.docx]

## Supplementary Figures and Tables

**Table S1:** List of search terms related to HCP points of interest

| List 1 | Professions | Anesthesiologist Assistant, Cardiovascular Technologist, Dialysis Technician, Emergency Medical Technician, Flight Nurse, Medical Laboratory Technician, Midwife, Nurse Anesthetist, Nurse Practitioner, Paramedic, Pharmacist, Phlebotomist, Physical Therapist, Physician  Assistant, Radiation Therapist, Respiratory Therapist |
| --- | --- | --- |
|  | Specializations | Anesthesiology, Critical Care, Dermatology, Dermatopathology, Emergency Medicine, Family Medicine, Gynecology, Hepatology, Immunology, Internal Medicine, Neurology, Obstetrics, OB/GYN, Oncology, Ophthalmology, Pathology, Pediatrics, Pharmacy, Psychiatry, Radiology, Surgery, Urology, General Practitioner |
| List 2 | POI types | Journal, Conference, Exhibit, Registration, Union, Council, Organization |
|  | English speaking countries | American, US, Canada, UK, England, Scotland, Wales, Northern Ireland, Ireland, South Africa, Australia, New Zealand |

**Table S2:** HCP points of interest in Twitter

| Role | #Accounts |
| --- | --- |
| Pharmacist | 130 |
| Nurse | 123 |
| Medical Doctors | 54 |
| Other medical professionals | 20 |
| Not related to a specific profession | 236 |
| Total | 563 |


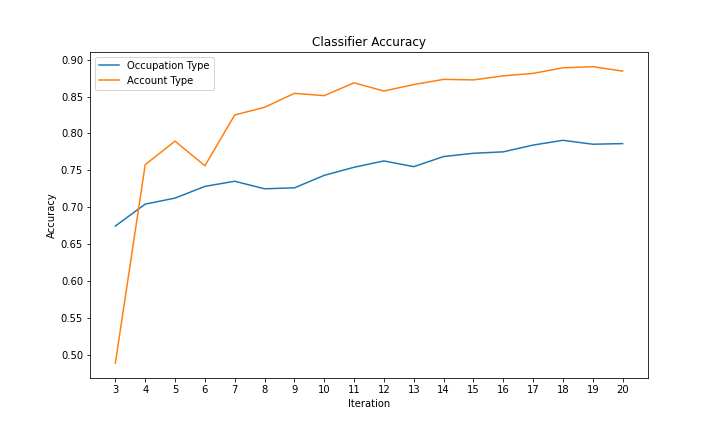
**Figure S1:** Health care professional classifier performance over iterations 3 to 20.

**Table S3:** COVID-19 terms removed during preprocessing

| Terms | coronavirusoutbreak, wuhancoronavirus, 2019ncov, ncov2019, wuhanvirus, covid2019, coronaviruscanada, covid, sarscov2, coronarvirus, coronarovirus, viruscorona, coronavid19, covid19usa, coronovirus, wuhancoronavirusoutbreak, coronavirusindia, coronavirusuk, coronaviruswuhan, chinacoronavirus, coronavirususa, covid19, coronaoutbreak, ncov19, virus, corona, 2019nCoV, COVID19, coronavirus, COVID-19, Coronavirustruth, CoronavirusOutbreak, coronaviruschina, coronavirusaustralia, CoronaVirusCanada, Wuhan, chinavirus, WuhanCoronavirus, nCoV2019, 2019-nCoV, nCoV, MERS-CoV |
| --- | --- |


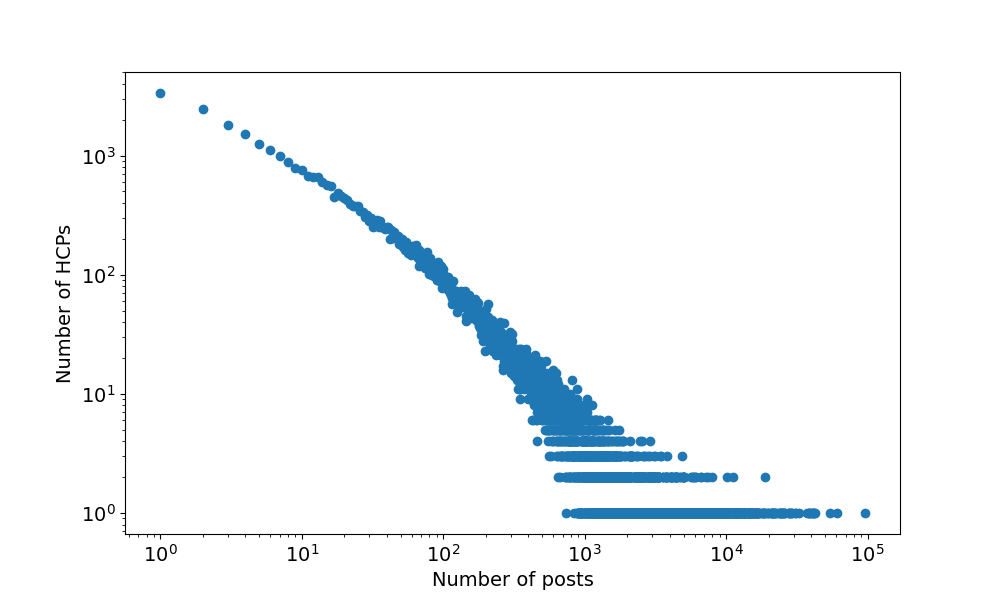


**Figure S2:** Distribution of health care professionals' (HCPs) tweeting activity.

**Table S4:** Distribution of topics across top 60 publishers and across the rest of the HCP population.

|  | Top 60 authors’ tweets | The rest |
| --- | --- | --- |
| Topic #0 | 25.16% | 25.98% |
| Topic #1 | 27.09% | 27.77% |
| Topic #2 | 19.4% | 12.84% |
| Topic #3 | 10.66% | 6.03% |
| Topic #6 | 3.6% | 10.49% |
| Topic #8 | 5.09% | 7.77% |
| Topic #9 | 0.74% | 1.03% |
| Topic #10 | 1.91% | 1.23% |
| Topic #16 | 1.41% | 1.84% |
| Other | 4.94% | 5.01% |


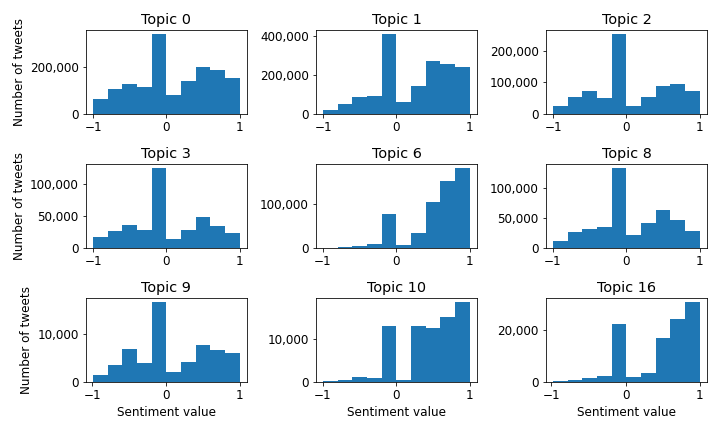


**Figure S3:** The sentiment distributions for each topic. These distributions are bimodal in case all the tweets with a sentiment value of zero were removed.
